# Muscle Recovery after a Single Bout of Functional Fitness Training

**DOI:** 10.3390/ijerph18126634

**Published:** 2021-06-20

**Authors:** Pablo García-Fernández, Eduardo Cimadevilla, Jesús Guodemar-Pérez, Ana María Cañuelo-Márquez, Juan Ramón Heredia-Elvar, Tomás Fernández-Rodríguez, María del Carmen Lozano-Estevan, Juan Pablo Hervás-Pérez, María Aránzazu Sánchez-Calabuig, Manuel Vicente Garnacho-Castaño, Juan Hernández Lougedo, José Luis Maté-Muñoz

**Affiliations:** 1 Department of Radiology, Rehabilitation and Physiotherapy, Complutense University of Madrid, 28040 Madrid, Spain; pablga25@ucm.es; 2 Department of Physiotherapy, Faculty of Health Sciences, Camilo José Cela University, 28692 Madrid, Spain; ecimadevilla@ucjc.edu (E.C.); jguodemar@ucjc.edu (J.G.-P.); tfernandez@ucjc.edu (T.F.-R.); jphervas@ucjc.edu (J.P.H.-P.); 3 Psychiatric Unit, Puerta de Hierro Hospital, 28222 Madrid, Spain; anamaria.canuelo@salud.madrid.org; 4 Department of Physical Activity and Sports Science, Alfonso X El Sabio University, 28691 Madrid, Spain; jelvaher@uax.es (J.R.H.-E.); jhernlou@uax.es (J.H.L.); 5 Department of Pharmacy, Faculty of Health Sciences, Alfonso X El Sabio University, 28691 Madrid, Spain; mloza@myuax.com (M.d.C.L.-E.); calabuig@uax.es (M.A.S.-C.); 6 Department of Physical Activity and Sport Sciences, TecnoCampus, College of Health Sciences, Pompeu Fabra University, 08302 Barcelona, Spain; mgarnacho@escs.tecnocampus.cat

**Keywords:** muscular fatigue, countermovement jump, CrossFit^®^, muscle strength, high-intensity training, lactate metabolism

## Abstract

Background: Functional fitness training (FFT) is a new exercise modality that targets functional multi-joint actions via both muscle-strengthening exercises and aerobic training intervals. The aim of the study was to examine muscle recovery over a 20 min period after an FFT workout in trained adults. Materials and methods: Participants were 28 healthy trained subjects. In a single session, a countermovement jump (CMJ) was performed to determine several mechanical variables (jump height, maximum velocity, power) before (preFFT) and 4, 10, and 20 min after the FFT workout (postFFT). In parallel, capillary blood lactate concentrations were measured pre- and 3 min postFFT. Heart rate was also measured before and after the workout, and perceived exertion was measured postFFT. Results: Significant differences between the time points preFFT and 4 min and 10 min postFFT, respectively, were produced in jump height (*p* = 0.022, *p* = 0.034), maximum velocity (*p* = 0.016, *p* = 0.005), average power relative (*p* = 0.018, *p* = 0.049), and average power total (*p* = 0.025, *p* = 0.049). No differences were observed in any of the variables recorded preFFT and 20 min postFFT. Conclusions: While mechanical variables indicating muscle fatigue were reduced 4 and 10 min postFFT, pre-exercise jump ability only really started to recover 20 min after FFT although not reaching pre-exercise levels. This means that ideally intervals of around 20 min of rest should be implemented between training bouts.

## 1. Introduction

Functional fitness training (FFT) is a new exercise modality that targets functional multi-joint actions via both muscle-strengthening exercises and aerobic training intervals [1]. CrossFit^®^ training is a form of FFT [2]. While there are many interpretations and descriptions that can be used to describe functional exercises, it has been proposed that these should consist of whole-body motor recruitment sequences in multiple movement planes such as vertical jumps, cleans, deadlifts, pull-ups, snatches, squats, etc. [3,4,5]. In classic training programs, these exercises of gymnastics and weightlifting are conducted as sets and repetitions along with long rest intervals and do not pursue significant cardiovascular responses or adaptations [6]. However, the prescription of these exercises as a circuit or intervals performed at high intensity targets muscle strength and power improvements as well as cardiovascular adaptations [2]. Accordingly, FFT programs involve a great variety of high-intensity functional movements designed to improve general measures of physical fitness (strength, cardiovascular endurance, flexibility, body composition, etc.) [2].

While high-intensity interval training (HIIT) is also defined by brief periods of high-intensity exercises separated by phases of rest or low-intensity exercise (FFT workouts lack a predefined rest interval), the movement patterns used in HIIT exercises are “unimodal” (such as running, pedaling, rowing, etc.) while FFT exercise is based on “multimodal” functional exercises [2].

Exercise-induced muscular fatigue is described as the reversible decline of muscle strength (muscle contractibility) over time (known as peripheral muscle fatigue during exercise) [7,8]. This reduced strength production during exercise may be considered a protection mechanism, as in its absence or when delayed, muscle cells and support tissues are susceptible to structural damage [9]. An indirect biomarker of muscle fatigue is blood lactate concentration [9]. Raised blood lactate concentrations coincide with cellular acidosis, and although this relationship should not be interpreted as cause–effect [10], this situation leads to diminished muscle contractile capacity [11].

Determining muscular fatigue through mechanical variables, rather than defects related to metabolism, reflects impaired muscle contractile properties or neuromuscular control [12]. One of the techniques most used to quantify neuromuscular fatigue through mechanical variables is to assess the loss of muscle capacity to develop force when performing a countermovement jump (CMJ) [13,14]. Muscular fatigue measured through the CMJ test involving muscle stretch-shortening cycles, reflects a reduction in muscle-tendon stiffness induced by structural damage to tendon insertions impairing CMJ performance [15,16,17].

To date, few studies have measured muscular fatigue levels in the different high-intensity exercise formats described. Muscular fatigue has been recently addressed in CrossFit^®^ [18,19]. This is an important parameter to consider due to the high intensity and high levels of power generated in this training modality. In this last study, elevated muscle fatigue was found pre–post workout of the day (WOD) [19], and also among the time points pre-WOD, intra-WOD, and post-WOD [18]. However, these studies were not designed to measure jump performance just minutes after exercise. Knowing for how long elevated fatigue levels are maintained in this type of high-intensity exercise could be an important marker of this variable, as recovery from fatigue is described as the rate at which muscle function returns to prefatigue levels [9]. The aim of this study was to examine muscle recovery in trained subjects minutes after a single FFT exercise workout. Knowing the kinetics of muscle recovery will give coaches and athletes an idea of the fatigue and stress generated by this form of exercise and, thus, help guide the design of effective training programs.

## 2. Materials and Methods

### 2.1. Experimental Design

**Figure 1:**
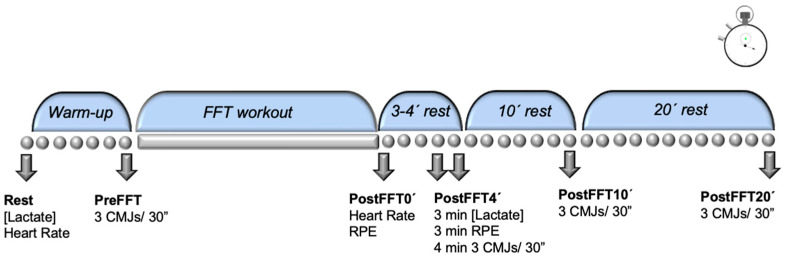
Study design.

Participants executed an FFT workout in a single session. Jump ability was calculated in a CMJ test before and after the workout at the time points: preFFT, 4 min postFFT (PostFFT4′), 10 min postFFT (PostFFT10′), and 20 min postFFT (PostFFT20′). Blood lactate concentrations were measured preFFT and 3 min postFFT, as it has been established that 3–5 min are necessary to resynthesize phosphocreatine deposits [20], thus allowing for better assessment of impacts on the glycolytic pathway once high-energy phosphagen deposits had been largely resynthesized. In addition, heart rate was measured before the warm-up and immediately after the workout, and perceived exertion was measured after the workout (Figure 1).

### 2.2. Subjects

The participants, 4 women and 24 men (age 28.70 ± 6.44 years, height 174.21 ± 8.70 cm, weight 75.18 ± 10.97 kg and body mass index 24.62 ± 1.86 kg·m^2^) undertook an FFT workout in a gymnasium equipped for this training modality. Participants were required to have more than 18 months of strength training experience including Olympic movements and free-weight in their routine workouts. All subjects were able to correctly execute a power clean (men 50 kg, women 35 kg) in addition to 15 successive pull-ups. Over the 3-month period leading up to the study, subjects were instructed not to take narcotic and/or psychotropic substances, stimulants, nutrient supplements, or performance-enhancing drugs. Moreover, exclusion criteria were neurologic, lung, metabolic, and cardiovascular diseases or any orthopedic limitation that could restrict their performance or the correct execution of the workout exercises. No participant was an elite athlete. Sample size calculation was based on the results of a pilot study with the same study protocol involving 10 sport science students. The calculation was performed with an α = 0.05 (5% chance of type I error) and 1 − β = 0.80 (power 80%) and by applying the results of previous studies, in which the sample size was the same or smaller. The calculated sample size was 28 strength-trained subjects.

Before the study outset, participants were informed about the study design and tests and exercises involved before obtaining their written informed consent. The study protocol was authorized by our university’s ethics commission (18 November 2019) according to the tenets of the Helsinki Declaration [21].

### 2.3. Functional Fitness Training (FFT) Workout

The workout began with a general warm-up of 5 min of rowing at a light–moderate intensity selected by each participant and 5 min of stretching and joint movements (active mobility exercises with complete movement ranges for the main joint nuclei protocolized and guided by a professional qualified in sports science). After the general warm-up, a specific FFT warm-up followed consisting of 6 reps each of burpees, box jumps, and bear crawls; 8 reps of slam balls (5–10 kg); and 10 reps each of in and out agility ladder and medicine ball on chest sit-ups (5–10 kg).

Once the general and specific warm-ups were completed, participants undertook a “rounds for time” all-out type of FFT work out. This consisted of 2 rounds of 6 power cleans (men 50 kg/women 35 kg), 10 object over shoulder (men 20 kg/women 15 kg), 14 wall ball shots (men 9 kg/women 7 kg), 18 shoulder to overhead dumbbell (DB) movements (men 20 kg/women 10 kg), and a 200 m run. Once participants finished these two rounds, they continued with a further two rounds of 6 pull-ups, 10 bodyweight squats, 14 DB power snatches (men 20 kg/women 10 kg), 18 box jumps (men 60 cm/women 50 cm), and a 100 m run (Table 1).

The exercises for this FFT workout were based on the movement standards for 2019 set by the International Functional Fitness Federation (iF3) (https://functionalfitness.sport/wp-content/uploads/2021/01/2021_iF3_Movement_Standards.pdf, accessed on 24 May 2021). This document describes the exercises included in iF3-authorized events in 2021 to standardize athletic tasks. According to these guidelines, two of the investigators with FFT experience supervised each participant.

### 2.4. Heart Rate

Before, during, and immediately after the FFT workout, heart rate (HR) was measured using a HR monitor (Polar RS-800CX; Polar Electro OY, Kempele, Finland). Heart rate data were collected and later extracted through the Polar Pro-Trainer 5 software.

### 2.5. Blood Lactate

Before the warm-up and 3 min after completing the FFT workout, index finger prick blood samples (5 µL) were measured to determine blood lactate concentrations in capillary blood with a previously validated and calibrated a portable analyzer (Lactate Pro LT-1710, Arkray Factory Inc., KDK Corporation, Siga, Japan) [22,23].

### 2.6. Rating of Perceived Exertion

Each participant graded their level of exertion on a Borg scale [24] from 6 to 20 as the rating of perceived exertion (RPE) from “very very light” to “maximum exertion”. RPEs were recorded at the levels cardiorespiratory (cardio), muscular, and general immediately after completing the workout and at 3 min postFFT [19,25]. To do this, each participant was requested to indicate with their finger on a scale of size DIN-A3, how hard they found the workout in terms of cardiorespiratory effort needed (RPEcardio), muscular effort (RPEmuscular), and in general terms (RPEgeneral). Before starting the FFTs, it was checked that each participant understood the meaning of each RPE. Thus, for RPEcardio, they were instructed to consider how fast their heart was beating and how out of breath they felt. For RPEmuscular, they were asked to assess how tired and strained their leg and arm muscles felt. To assess RPEgeneral, subjects were asked to describe how hard their workout felt overall. Participants were instructed to avoid verbal descriptions and to just point directly to the appropriate place on the scale with their finger.

### 2.7. Muscular Fatigue

Muscular fatigue was assessed in the legs by measuring vertical reaction forces (0–10 kN; sampling velocity 0.5 kHz) in a CMJ [26] performed on a portable force platform of 92 × 92 × 12.5 cm (Quattro Jump model 9290AD; Kistler Instruments, Winterthur, Switzerland) before the workout (preFFT) and PostFFT4′, PostFFT10′, and PostFFT20′. After the warm-up before starting the FFT workout, subjects stood on the platform with their legs extended and hands on hips. The legs were first flexed to 90 degrees (eccentric action) and then extended explosively in a coordinated way (concentric action) targeting maximum height. When off the ground, the subject extended the knees. To complete the jump, the ground was touched with the toes first. Participants were requested to avoid sideways displacements and to keep their hands on their hips during this test. After finishing the FFT workout, participants were encouraged to keep moving in the cool down while they waited to do the CMJ tests [27,28].

At each time point, individuals completed two jumps separated by 30 s and the means were recorded of maximum take-off velocity, jump height, first force peak maximum, average power total, average power relative, peak power total, and peak power relative. Losses in jump height and power during an exercise session are defined as indicators of mechanical and neuromuscular fatigue, along with metabolic stress [13]. The force platform was connected to a PC and the software package Kistler (Quattro Jump software, version 1.1.1.4, Kistler Instruments, Winterthur, Switzerland) was used to quantify the different variables. The vertical ground reaction force (GRF) data were obtained during the jump (range 0–10 kN; sampling frequency 0.5 kHz). To determine the vertical component of the center of mass (COM) velocity, the impulse method was used [29]. To record net impulse, the GRF was integrated from two seconds before the subject’s first movement [30]. COM vertical velocity was given by dividing net impulse by the subject’s weight [31]. Maximum velocity reached at the end of the concentric muscle action of the jump was considered as maximum take-off velocity (V_max_). Jump height (cm) was calculated from V_max_ of the COM and the deceleration of gravity. Height = ((V_max_)2/2 × 9.81). Power was calculated from the unfiltered force–time history using the impulse momentum principle [32]. Mean relative power (watts·kg^−1^) was determined as the product of mean velocity and the vertical component of the ground reaction force.

Other variables used to evaluate jump performance and mechanical neuromuscular fatigue, jump duration, eccentric phase (ECC) duration, isometric phase (ISO) duration, and concentric phase (CON) duration [33,34] were also determined. This was done by measuring the different vertical reaction forces during the CMJ (range 0–10 kN; sampling velocity 0.5 kHz) according to the method described by Maté-Muñoz et al. [18].

### 2.8. Statistical Analysis

The Shapiro–Wilk’s test was utilized to check the normal distribution of data. To measure muscular fatigue produced during the FFT workout, a repeated measures linear model for the factor Time was constructed and contrasted with the Mauchly sphericity test. When the hypothesis of sphericity was discarded, we used the univariate F statistic, adjusting it with the Greenhouse–Geisser correction index. When significant differences were detected between measures, the post hoc Bonferroni test was implemented.

To detect differences between RPE at the different time points, the t test for related measures was used. Relationships between metabolic responses and jump ability loss post-exercise and FFT workout duration were assessed through Pearson correlation.

The general linear model procedure generates an effect size known as partial eta-squared, which is classified into small = 0.01, medium = 0.06, and large = 0.14 [35]. All data are provided as their means (M), standard deviations (SD), and 95% confidence intervals (CI). In addition, the effect size (ηp^2^) and statistical power (SP) of the data were determined. Percentages of improvement were calculated with the equation ((post − pre)/pre × 100). Significance was set at P < 0.05. All statistical tests were performed using the software package SPSS version 25.0 (SPSS Inc., Chicago, IL, USA).

## 3. Results

FFT duration was 12.63 ± 2.99 min. Mean HR for the FFT workout was 171.52 ± 9.76 bpm (91% HRmax). The results obtained for RPE are shown in Table 2. The high metabolic stress of the FFT workout was confirmed by a [lactate] of 15.23 ± 3.58 mmol·L^−1^ and a final HR of 181.81 ± 8.20 bpm. From the end of the FFT workout to 3 min later, RPEmuscular and RPEcardio fell by ~20% and RPEgeneral by ~18%.

The jump ability data for the different time points of the FFT workout (Table 3) indicate significant reductions in jump height ((3,81) = 4.557; *p* = 0.005), maximum jump velocity (F(3,81) = 5.840; *p* = 0.001), average power total (F(3,81) = 3.444; *p* = 0.021), total jump duration (F(3,81) = 4.548; *p* = 0.005), and CON duration (F(3,81) = 6.949; *p* = 0.000). In our post hoc analysis using the Bonferroni index comparing pre- and postFFT workout measures, significant differences were detected for preFFT versus PostFFT4′ (jump height *p* = 0.022, maximum velocity *p* = 0.016, average power relative *p* = 0.018, average power total *p* = 0.025, and CON duration *p* = 0.002); for preFFT versus PostFFT10′ (jump height *p* = 0.034, maximum velocity *p* = 0.005, average power relative *p* = 0.049, average power total *p* = 0.049, total jump duration *p* = 0.037, and CON duration *p* = 0.004); and for preFFT versus PostFFT20′ (total jump duration *p* = 0.018, CON duration *p* = 0.006). In other words, in relation to preFFT values, at PostFFT4′, a 6% loss of jump height was produced, which increased at PostFFT10′ to 6.8% and fell at PostFFT20′ to 4.4%, although no significant differences were found between preFFT and PostFFT20′ (*p* = 0.561). This jump height loss 20 min after the workout was also observed in the variable maximum jump velocity (losses were 2.4%, 3.2%, and 2% at the 3 time points, respectively), without significant differences detected between preFFT and PostFF20′ (*p* = 0.151). However, losses in average power relative and total peaked at 4 min post exercise (~3.7%), and then fell to ~2.9% at PostFFT10′ and to ~1.1–1.4% at PostFFT20′ (not being significant between preFFT and PostFFT20, *p* = 1.000). Jump duration shortened throughout the time of recovery (PostFFT4′ 2.9%, PostFFT10′ 5.4%, PostFFT20′ 6.8%). Among the different jump phases, the CON phase underwent a significant decrease after the workout (PostFFT4′ ~15%, PostFFT10′ ~19%, PostFFT20′ ~17%) (Table 3).

**Figure 2:**
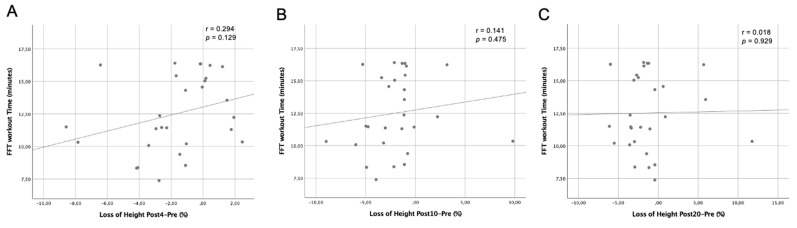
Correlation between FFT workout duration and loss of jump height in minutes 4 (**A**), 10 (**B**), and 20 (**C**) after the workout.

Figure 2 shows the relationship between the time needed to complete the FFT workout and the loss produced in jump height at postFFT minutes 4 (Figure 2A), 10 (Figure 2B), and 20 (Figure 2C). Weak associations (|r| < 0.30) were observed in all the jump measures after the FFT workout, likely attributable to a random effect as the relationships were not significant (A: r = 0.294, *p* = 0.129, B: r = 0.141, *p* = 0.475, C: r = 0.018, *p* = 0.929). Neither were significant relationships found between [lactate] post-exercise and FFT workout duration (r = −0.083, *p* = 0.681). Furthermore, although moderate correlation was observed between metabolic responses and jump ability loss, this was not significant (r = −0.360, *p* = 0.065).

## 4. Discussion

This study examines jump ability in a countermovement jump test after executing a single FFT workout in a group of subjects with functional training experience. According to our heart rate data, the FFT workout executed by the 28 study participants can be described as an exercise intensity of 91% considering HR_max_ = 208 − 0.7 × age [36] (mean HR = 171.52 ± 9.76 bpm).

Our main finding was that, while similar reductions were produced in mechanical variables at the three time points post-exercise (PostFFT4′, PosFFT10′, PostFFT20′), pre-exercise jump ability starts to recover 20 min after the workout. This means that while there is a tendency toward the recovery of neuromuscular capacity after 20 min, fatigue is still considerable and affects jump ability. Thus, significant differences were observed in jump height, maximum velocity, and average power (relative and total) between minutes 4 and 10 postFFT, whereas by minute 20 postFFT, these measures had started to recover approaching baseline values. These data are interesting as they allow us to identify the approximate time point at which jump variables start to recover. Measurements started 3 min after the workout had been completed, when general RPE values were around 13. As also observed by Alberton et al. (2013) [37], the intensity of exertion perceived by our subjects fell until it was above the first ventilatory threshold. RPEmuscular ratings were higher than RPEcardio and RPEgeneral. This could be explained by the characteristics of this type of work and these differences could indicate that peripheral (neuromuscular) factors are more determining and limiting for performance than central (cardiorespiratory) factors. At this time, the loss of PostFFT4′ jump height relative to its preFFT value was 6%. Maté-Muñoz et al. (2017) [18] reported similar jump ability losses after executing gymnastics and weightlifting CrossFit^®^ WODs (of 6.5% and 7.4%, respectively), while this loss in response to the metabolic conditioning WOD was much lower (3.6%), likely because of rest periods between sessions. In the second CMJ 6 min later (PostFFT10′), the jump height loss recorded was 6.8% compared to baseline. However, 20 min after the FFT workout, jump ability variables failed to differ significantly from pre-exercise levels (*p* > 0.05) and almost 2.5% of the greatest jump height loss was recovered (PostFFT10′ = 6.8% versus PostFFT20′ = 4.4%). Other variables showed similar behavior such as maximum jump velocity and average power both absolute and relative to bodyweight. Hence, from its preFFT value, maximum velocity had fallen by 2.4% at PostFFT4′ and by 3.2% at PostFFT10′. In addition, this variable showed a 2% reduction at PostFFT10′ with respect to PostFFT20′, such that no significant difference in velocity was produced between preFFT and PostFFT20′. In other words, 1.2% of jump velocity was recovered between minute 10 and minute 20 after the workout. Maximum velocity losses between preFFT and PostFFT4′ reported in the literature are similar for the CrossFit^®^ WODs “Cindy” and “power clean” (2.7% and 3.1%, respectively), while for the skip rope “double unders” WOD, jump velocity loss was half this amount (1.2%), probably because of the “Tabata” method employed in this WOD [18]. Here, we also observed losses in average power generated in the CMJ. At PostFFT4′ a loss of around 3.75% was recorded. Subsequently, values recovered at PostFFT20′ compared to PostFFT4′ by 2.3% (average power total) and by 2.6% (average power relative), with no significant differences noted between preFFT and PostFFT20′ values. Similar average power relative data to those obtained here at PostFFT4′ were detected for the “Cindy” CrossFit^®^ WOD (~−4%), while power losses reported for the metabolic conditioning (~2%) and weightlifting WODs (7.35%) were lower and higher, respectively [18]. In summary, the variables jump height, maximum take-off velocity, and power were reduced by the same percentages at 4 min post-exercise as observed in other studies for gymnastics and weightlifting CrossFit^®^ WODs. Performance losses are lower in metabolic conditioning exercises because of scheduled breaks.

Some authors who have used jump duration as a measure of mechanical muscular fatigue [34,38] report that total CMJ duration is significantly shorter than its preFFT value, with increasing recovery time. Thus, the CMJ took less time to execute at 20 min (−6.84%) than at 10 or 4 min after the workout (−5.39% and −2.89%, respectively), compared to at the start of the session, with no significant differences detected between these values. Thus, total CMJ durations after the workout were similar, and temporal jump execution variables were also similar as significant differences were also not detected following exercise in the duration of each of the jump’s stages (ECC, ISO, and CON).

However, we observed that while 20 min after conducting the FFT workout jump ability variables had not returned to their pre-exercise values, significant differences were not observed between preFFT and PostFFT20′ as produced between PostFFT4′ and PostFFT10′. This start in the recovery of jump ability 20 min after exercise could be the outcome of two factors, a metabolic and a mechanical one.

### 4.1. Metabolic Factors

At the metabolic level, while it has been established that depleted phosphocreatine stores determine a reduction in jump capacity [39], our first measurements were made at PostFFT4′, which is more than sufficient time to recover phosphocreatine levels of around 100% [20]. However, the use of anaerobic glycolysis and consequent changes in the intramuscular environment was reflected by the high capillary blood lactate levels detected 3 min after finishing the FFT workout (15.23 ± 3.58 mmol·L^−1^). Compared to the lactate concentrations elicited by similar training protocols, our values were much higher. Thereby, we previously observed capillary blood lactate levels of ~12 mmol·L^−1^, ~10 mmol·L^−1^, ~11 mmol·L^−1^, respectively, in two CrossFit^®^ studies [18,19] for WODs “Cindy” and “Tabata”, skip rope, “double unders”, and “power cleans”. These power cleans were executed for 5 min at 40% maximal strength, as recorded in a maximum repetition (1RM). In another study, a blood lactate concentration of 14.5 mmol·L^−1^ was reported after a “Cindy” protocol. This value could be an overestimate as the resting levels reported by its authors were high at around 4 mmol·L^−1^ [40]. Metabolic acidosis takes place when an increase is produced in anaerobic glycolysis when type II motor units are recruited [41]. These motor units are less oxidative than type I [42]. As type II motor units feature a faster contraction velocity [43], the workload planned in our FFT workout and the need to complete the workout as quickly as possible, may have been a determining factor for the preferential recruitment of type II motor units. In turn, these type II motor units are more fragile than type I and more dependent on a glycolytic metabolism [43]. However, after 20 min of recovery, it could be that lactate clearance could outstrip its production, reducing lactate levels and diminishing the buildup of hydrogen, which would raise the pH. This means that metabolic acidosis could diminish, in turn, recovering motor type II units, thus improving values of jump height, maximum velocity, and average power. Unfortunately, we can only speculate about a possible reduction in blood lactate at 20 min, as at this time point capillary blood samples were not taken. This can be considered a limitation of our study. Consistent with reduced blood lactate levels during the recovery period, studies such as that by Lucertini et al. (2017) [44] in which lactate was measured (8 measures, one every 4 min) after a 30 s anaerobic power test (Wingate), detected a 19 min posttest blood lactate level of ~3 mmol·L^−1^, if recovery was active, and one of ~7 mmol·L^−1^, if passive. In the same line of investigation, Franchini et al. (2009) [45] examined active and passive rest intervals up to 15 min after a judo combat. Blood lactate concentrations after 15 min of active and passive recovery were 4.1–5.3 and 6.4–7.3 mmol·L^−1^, respectively. In another study that conducted a fatigue protocol until exhaustion, stretch-shortening cycle exercise the blood lactate concentrations were 7.1 mmol·L^−1^ at 3 min, 7.2 mmol·L^−1^ at 5 min, and 2.7 mmol·L^−1^ at 30 min after exercise [15]. In our study, we encouraged participants to move around the gym while waiting to undertake the CMJ tests after the FFT workout. Hence, based on the lactate levels recorded during active recovery in both these studies, at 20 min, blood lactate could have dropped sufficiently to eliminate a large part of metabolic acidosis and, thus, promote the recruitment of more type II motor units, generating greater contraction forces to recover measures of jump ability. Notwithstanding, it is important to understand that these types of exercise induce significant stress levels in terms of demands on the glycolytic pathway, such that a certain recovery time is needed. We should also consider that currently, these and similar models are used consecutively over several days a week in all types of individuals.

### 4.2. Mechanical Factors

Another factor indicating the start of the recovery of jump capacity would be changes in muscle-tendon stiffness. Stiffness represents the degree of opposition to deformation by a biological tissue, and this stiffness has been shown to diminish as a consequence of fatigue or in response an intense stretch-shortening cycle exercises [15,16,17]. Because of the high intensity of the FFT workout in which many exercises involved the lower limbs, it is likely that muscle-tendon stiffness was reduced. Nevertheless, a recent study confirmed that decreased joint stiffness was due to reductions in active muscle stiffness after repeated hopping exercises, whereas tendon stiffness did not change [46]. In addition, another study found no changes in either stiffness or initial length of the Achilles tendon after a single period of high-impact exercise developed until exhaustion, while the maximum isometric plantar flexion force was significantly reduced (20%) [47]. Neither were stiffness changes produced in Achilles tendon stiffness after 12 healthy individuals participated in a marathon [48]. A greater stiffness may modify the mechanical properties of the muscle and tendon and could provoke overuse injuries as observed in sprint track cyclists [49]. Recently, Klich et al. (2020) [49] reported tendon and patellar tendon thickening and stiffening in endurance and sprint track cyclists. In addition, the latter cyclists showed a greater tendon thickness before the race than the endurance track cyclists, suggesting quadriceps muscle edema and hypervascularity [49]. Moreover, the sprint track cyclists also experienced a greater change in post- versus pre-exercise stiffness than the endurance track athletes [49]. Moreover, reduced muscle-joint stiffness has been associated with fatigue generated by cyclic muscle contractions in the quadriceps [50]. In our study, after 20 min of recovery, perhaps the increase in muscle-tendon stiffness facilitates the recovery of jump ability. Previous studies have confirmed that increased stiffness serves as a mechanism potentiating the muscle stretch-shortening cycle [51], this being the muscle action conducted in the CMJ test used to measure muscular fatigue. When the concentric phase was examined, significant differences were noted in PostFFT4′ (−14.6%), PostFFT10′ (−18.7%), and PostFFT20′ (−17%) versus preFFT durations. That is, although all values were similarly reduced (by some 14% to 19%), the greatest reduction in concentric phase duration was observed after 10 min of recovery. This coincides with the greater losses produced in jump height, maximum velocity, and average power. However, at PostFFT20′ jump height, maximum velocity, and average power were higher than at PostFFT4′ and PostFFT10′. Accordingly, the recovery of mechanical jump variables could be explained by a greater muscle-tendon stiffness. Further, as commented above, this recovery may also be attributed to a reduction in metabolic stress and the start of the use of type II motor units. Further work is needed to understand the acute fatigue mechanisms elicited by exercises that generate intense effort in relation to changes in tendon stiffness, muscle tension, and metabolic factors, inducing reduced performance.

Finally, no significant correlation was observed between FFT workout duration and jump height loss during the recovery period. With this analysis, we tried to establish an adequate recovery regimen among the participants who more rapidly completed the FFT workout. As all our subjects had training experience with this form of exercise, it could be that although workout duration varied from 7.39 to 16.40 min, recovery was uniform irrespective of workout duration. Similarly, there is also no relationship between [lactate] post-exercise and FFT workout duration. More work is needed to compare recovery times between trained and untrained subjects following FFT protocols in terms of jump ability measures and blood lactate responses.

These results are consistent with those of others such as Tibana et al. (2018) [52] and Falk Neto et al. (2019) [53], in which some of the variables examined in the present study were controlled in FFT workouts of different durations, and similar conclusions regarding high metabolic and fatigue stress were found. This is useful for scheduling training as this type of workout on consecutive days and even at a high weekly frequency might not be adequate due to the need to ensure sufficient levels of recovery.

It would also be of interest to more precisely define optimal workloads for these training models. This is because the similar absolute loads lifted (kg) and number of repetitions executed would suggest differences in relative work intensities attained by each participant and, thus, different extents of fatigue produced in each set and exercise. This, along with the different rest periods associated with these efforts (density), could indicate that these training models generate intense fatigue during a workout irrespective of variations in degrees of effort and workout durations among the participants. This is characteristic of this type of workout and should be considered by coaches when designing their training programs.

### 4.3. Limitations

Due to the characteristics of the exercises, tests for the upper limbs could have been included, such as changes in speed when loading 1 m·s^−1^ in pushing or pulling actions of the upper limbs (bench press or row, for example). The complexity of this study made the researchers select tests showing the greatest influence on functional capacity such as lower-limb strength. Moreover, the 10 min difference between PostFFT10′ and PostFFT20′ should be considered. That is, the start in the recovery of jump ability could be at intermediate time points (e.g., PostFFT12′ or PostFFT17′). Studies are, therefore, needed to pinpoint the exact time point when jump ability starts to recover. A further limitation that needs to be addressed in future studies is the small number of female participants of our study.

## 5. Conclusions

The FFT workout executed can be described as a high-intensity effort of 91% (mean HR = 171.52 ± 9.76 bpm), generating high metabolic stress ([lactate] = 15.23 ± 3.58 mmol·L^−1^) and a post-exercise RPE score between ~13–16, which is considered “hard” work.

Having assessed muscle recovery after a FFT workout in trained subjects through jump ability, our data reveal losses of jump height, maximum velocity, and average power at 4 and 10 min post-exercise compared to pre-exercise. These losses could start their recovery 20 min after participants completed the FFT workout, without yet returning to preFFT values. However, while jump ability started to recover (without reaching preFFT values at this point), similar temporal parameters of jump execution measured in the different jump stages (ECC, ISO, and CON) and total jump durations were observed. We propose that this start in the recovery process could be attributed both to a reduction in intramuscular metabolic acidosis allowing for the use of more type II motor units (metabolic factor) and to improved muscle-tendon stiffness (mechanical factor). More studies are, nevertheless, needed to understand these acute mechanisms of muscular fatigue, as this type of exercise generates intense fatigue, and this should be taken into account when designing training protocols.

## Figures and Tables

**Table 1:** FFT workout conducted by the participants of this study. Exercise descriptions taken from the 2021 movements standards set by the iF3.

Functional Fitness Training Workout			
**2 rounds (r) × (6 power clean)**		**2 r × (6 pull-up)**	
A power clean requires the athlete to catch the bar in the front rack position without reaching the bottom of a squat.	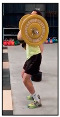	To complete the pull-up, the subject grips a suspended horizontal bar with both hands and fully extends both arms at the repetition bottom. At the repetition top, the chin should break the uppermost horizontal plane of the bar. A kip of any style may be used. The repetition is counted when the athlete’s chin breaks the top-most horizontal plane of the bar.	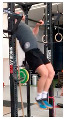
Men: 50 kg	Women: 35 kg		
**2 r × (10 object over shoulder)**		**2 r × (10 bodyweight squat)**	
For the object over shoulder, the athlete must pick up the designated object and toss or push it over the shoulder while the athlete reaches full extension of the hips. The athlete may lift the object using any technique.	* 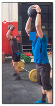 *	The bodyweight squat is a bodyweight movement where, the athlete begins in a standing position with an open hip angle, descends to a full squat, with the creases of both hips below the plane of the top of the knees, and returns to the standing position with hips returning to an open angle.	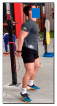
Men: 20 kg	Women: 15 kg		
**2 r × (14 wall ball shot)**		**2 r × (14 power snatch)**	
The wallball shot is performed with a medicine ball and an elevated target. With the medicine ball in the frontal plane, the athlete must descend to a bottom-of-squat position and then, while ascending, throw the ball so that it makes contact at or above a designated height. Jumping during the repetition is permissible but not required.	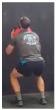	The power snatch requires the athlete to catch the dumbbell overhead with elbows fully extended but without achieving the bottom of a squat during the task.	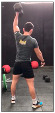
Men: 9 kg	Women: 7 kg	Men: 20 kg	Women: 10 kg
**2 r × (18 shoulder to overhead)**		**2 r × (18 box jump)**	
Shoulder to overhead movements involve elevating dumbells from a static position at the shoulder to a static position overhead. The athlete may use a single, simultaneous bending of the hips and/or knees to assist in elevating the dumbbells to the top of the repetition.	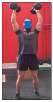	The box jump requires the athlete to initiate a jump from the ground with both feet simultaneously, land on top of a designated object (e.g., box) with both feet and demonstrate static control.	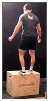
Men: 20 kg	Women: 10 kg	Men: 60 cm (23.6 inch)	Women: 50 cm (19.7 inch)
2 r × (200 m run)		2 r × (100 m run)	

**Table 2:** Ratings of perceived exertion recorded in the FFT workout.

Variable	Group	M ± SD	*t*	*p*
Final RPE Muscular	PostFFT0′	16.50 ± 2.43	6.02	0.000 *
	PostFFT3′	13.18 ± 3.30		
Final RPE Cardio	PostFFT0′	14.64 ± 2.75	6.32	0.000 *
	PostFFT3′	11.80 ± 2.40		
Final RPE General	PostFFT0′	15.67 ± 2.01	6.82	0.000 *
	PostFFT3′	12.83 ± 2.58		

RPE = rating of perceived exertion, FFT = functional fitness training, PostFFT0′ = 0 min after the FFT workout, PostFFT4′ = 4 min after the FFT workout; * = differences between groups; *p* < 0.01. M (mean) ± SD (standard deviation).

**Table 3:** Jump ability variables recorded preFFT and at postFFT minutes 4, 10, and 20.

Variable	Pre (M ± SD, 95% CI)	Post 4′ (M ± SD, 95% CI)	Post 10′ (M ± SD, 95% CI)	Post 20′ (M ± SD, 95% CI)	% Loss Pre–Post 4′	% Loss Pre–Post 10′	% Loss Pre–Post 20′	*p* for the Time Effect	η_p_^2^	SP
Jump height (cm)	28.13 ± 8.35 ^‡,^ * (24.89–31.36)	26.45 ± 7.45 (23.57–29.34)	26.22 ± 7.90 (23.16–29.28)	26.90 ± 7.64 (23.93–29.86)	−5.97	−6.79	−4.37	0.005 *	0.144	0.871
V_max_ (m·s^−1^)	2.52 ± 0.30 ^‡,^ * (2.40–2.64)	2.46 ± 0.31 (2.34–2.58)	2.44 ± 0.33 (2.32–2.57)	2.47 ± 0.31 (2.35–2.59)	−2.38	−3.17	−1.98	0.001 *	0.178	0.926
F_max_ (Newtons)	1849.92 ± 295.19 (1735.46–1964.39)	1864.41 ± 323.59 (1738.93–1989.88)	1883.60 ± 317.92 (1760.33–2006.88)	1825.58 ± 358.28 (1686.65–1964.50)	0.78	1.82	−1.31	0.213	0.055	0.342
APR (watts·kg^−1^)	26.50 ± 4.58 ^‡,^ * (24.73–28.28)	25.50 ± 4.70 (23.68–27.32)	25.73 ± 4.82 (23.86–27.60)	26.20 ± 4.69 (24.39–28.02)	−3.77	−2.91	−1.13	0.151	0.069	0.370
APT (watts)	2005.10 ± 458.11 ^‡,^ * (1827.45–2182.73)	1930.96 ± 465.85 (1750.32–2111.60)	1947.61 ± 464.40 (1767.54–2127.68)	1977.61 ± 466.68 (1796.65–2158.57)	−3.70	−2.87	−1.37	0.021 *	0.113	0.754
PPR (watts·kg^−1^)	48.54 ± 8.25 (45.34–51.74)	47.90 ± 8.45 (44.62–51.18)	47.62 ± 8.59 (44.29–50.95)	47.83 ± 8.04 (44.71–50.95)	−1.32	−1.90	−1.46	0.322	0.042	0.307
PPT (watts)	3672.85 ± 819.42 (3355.11–3990.58)	3629.43 ± 849.57 (3300.00–3958.86)	3607.57 ± 842.39 (3280.93–3934.22)	3624.46 ± 819.34 (3306.75–3942.17)	−1.18	−1.78	−1.32	0.384	0.037	0.270
Total jump duration (s)	0.760 ± 0.13 *^,†^ (0.708–0.812)	0.738 ± 0.13 (0.689–0.787)	0.719 ± 0.16 (0.656–0.782)	0.708 ± 0.13 (0.658–0.759)	−2.89	−5.39	−6.84	0.005 *	0.144	0.871
ECC duration (s)	0.577 ± 0.15 (0.519–0.634)	0.580 ± 0.17 (0.525–0.636)	0.567 ± 0.17 (0.501–0.636)	0.553 ± 0.14 (0.498–0.608)	0.52	−1.73	−4.16	0.323	0.042	0.306
ISO duration (s)	0.0126 ± 0.005 (0.011–0.015)	0.0113 ± 0.004 (0.010–0.013)	0.0111 ± 0.005 (0.009–0.013)	0.0139 ± 0.011 (0.010–0.018)	−10.32	−11.90	10.32	0.325	0.040	0.225
CON duration (s)	0.171 ± 0.06 ^‡,^ *^,^^†^ (0.146–0.195)	0.146 ± 0.06 (0.122–0.170)	0.139 ± 0.05 (0.118–0.160)	0.142 ± 0.06 (0.120–0.163)	−14.62	−18.71	−16.96	0.000 *	0.205	0.974

V_max_ = maximum jump velocity, F_max_ = maximum force, APR = average power relative, APT = average power total, PPR = peak power relative, PPT = peak power total, ECC = eccentric phase, ISO = isometric phase; CON = concentric phase, Pre = pre-exercise, Post’ = minutes after the FFT workout, cm = centimeters; m·s^−1^ = meters per second, kg = kilograms, s = seconds, ^‡^ = significant differences between Pre and Post 4′ (*p* < 0.05); * = significant differences between Pre and Post 10′ (*p* < 0.05); ^†^ = significant differences between Pre and Post 20′ (*p* < 0.05). Data expressed as the mean ± standard deviation (SD) ± 95% confidence interval (CI). η_p_^2^ = partial eta squared. SP = statistical power.
